# Evaluation of the (Baha) technique of scleral indentation using a self-retained scleral indenter during vitrectomy surgery: a randomized trial

**DOI:** 10.1007/s10792-024-03028-6

**Published:** 2024-02-17

**Authors:** Samir El Baha, Moutaz Ghandour, Islam S. H. Ahmed

**Affiliations:** 1 Ophthalmology Department, Faculty of Medicine, Alexandria University, Alexandria, Egypt; 2 Anaesthesia and Surgical Intensive Care Department, Faculty of Medicine, Alexandria University, Alexandria, Egypt; 3 Faculty of Medicine, Alexandria University, Khartoum Square, Azarita, Alexandria, Egypt

**Keywords:** Retinal detachment, Pars plana vitrectomy, Leyla retractor, Self-retained scleral indenter

## Abstract

**Aims:**

The current study compared a novel technique of scleral indentation using the self-retaining Leyla retractor to the conventional scleral self-indentation with the chandelier light.

**Methods:**

Patients with rhegmatogenous retinal detachment were randomized on a 1:1 basis to either have the (Baha) indentation using a tip of a thimble scleral indenter welded to the support for the Leyla retractor system or to have the conventional scleral indentation while using a 25-gauge chandelier light. A video was recorded for the surgery of all the cases and reviewed by another consultant masked to the type of indentation. The indentation duration (i.e., the time in seconds between the first appearance of a hump due to scleral indentation in the recorded video until its final disappearance) was measured for every case.

**Results:**

The current study included 60 eyes of 60 adults with a mean age of 59.6 ± 9.8 years. Thirty-nine of the eyes were phakic and 21 were pseudophakic. The mean indentation time was 618 ± 87 and 696 ± 72 s in (Baha) indentation and conventional indentation groups, respectively. The difference was not statistically significant (*p* = 38). There was a positive correlation between the vertical palpebral fissure height and the indentation duration for both (Baha) indentation (*r* = 0.58) and conventional indentation groups (*r* = 0.42). Readjustment of the chandelier endo-illumination was required in 19 cases (63.3%) in the conventional indentation group. Iatrogenic breaks or accidental crystalline lens touch did not occur in any case.

**Conclusion:**

The (Baha) technique is effective and safe, especially in patients with a larger palpebral fissure.

**Supplementary Information:**

The online version contains supplementary material available at 10.1007/s10792-024-03028-6.

## Introduction

Scleral indentation is essential to visualize and stabilize the peripheral retina during vitrectomy for rhegmatogenous retinal detachment (RRD). [1] This enables examination of the peripheral retina up to the ora serrata by pushing the peripheral structures into view and allows satisfactory removal of the peripheral vitreous at the vitreous base which is mandatory to improve the surgical outcome. [2, 3] Peripheral retinal lesions like retinal tears, holes, and areas of vitreoretinal traction can be better seen by alternating the angle of viewing to increase the contrast. [4].

However, a second hand during the surgery may be needed due to the possible distortion of the cornea, surgical field crowding, or perfluorocarbon liquids (PFCL) bubble accumulation. [1, 5].

The use of a self-retained chandelier endo-illumination with scleral self-indentation during peripheral

vitrectomy was reported in several studies to provide uniform illumination to improve visualization. [6–10] However, to increase the illumination over the area of interest during the surgery readjusting to chandelier light was often needed.

Prof. M. G. Yasargil designed a self-retaining brain retractor and named it after his daughter (The Leyla retractor). It has been used in brain surgery for brain retraction by applying an even, gentle pressure.

This retractor consists of three parts: a support for a retractor, a flexible arm, and a fixation base which directly attaches over the sterile drapes to the operating table. [11].

In the current study, we compared the results of a novel technique of scleral indentation; the (Baha) technique using the self-retaining Leyla retractor to the conventional self-indentation of the sclera with the use of the chandelier light during the vitrectomy surgery.

## Methods

The current study was a prospective randomized comparative interventional study in which we enrolled patients older than 18 years who presented with a rhegmatogenous retinal detachment to the Ophthalmology department of the Alexandria Main University Hospital in the period between February 2021 and February 2022 and requiring retinal detachment repair by pars plana vitrectomy (PPV) surgery.

The study protocol was approved by the Faculty of Medicine Ethics Committee, Alexandria University, Egypt. After explaining the procedures, an informed consent was signed by all the patients. The study was conducted in accordance with the tenets of the Declaration of Helsinki.

A vitreoretinal consultant (I. A.) who was masked to the procedure that would be done for the patient conducted a comprehensive ophthalmic examination for all the patients. Demographic data, and data about the preoperative best corrected visual acuity (BCVA), palpebral fissure height, lens status and the location of the primary retinal break were collected.

Next, the patients were randomized on a 1:1 basis by a research nurse into two groups using the closed envelope technique. Cases in the first group had the (Baha) technique of scleral indentation using the self-retaining Leyla retractor. On the other hand, cases in the second group had the conventional self-indentation of the sclera done while using a 25-gauge chandelier lighting system (Alcon Chandelier lighting system; Alcon).

All the surgeries were done by a single vitreoretinal surgeon (S. E.) under general anaesthesia. In all cases of both groups, a non-contact wide-angle viewing system (Resight; Carl Zeiss Meditec AG, Jena, Germany) and an Alcon Constellation vitrectomy machine (Alcon Laboratories, Fort Worth, TX) were used. In all cases, three valved Alcon 23-gauge trocar cannulas were inserted (inferotemporal for the infusion cannula, superotemporal and inferonasal) 3.5 mm from the limbus in pseudophakic or aphakic eyes or 4 mm from the limbus in phakic eyes. In addition, in the conventional indentation cases, a 25-gauge chandelier lighting system (Alcon Chandelier lighting system; Alcon) was inserted at the 6 o’clock position. Cataract removal—whenever indicated—was done using phacoemulsification with insertion of a foldable intraocular lens followed by suturing the main corneal incision with a single 10/0 nylon suture. The steps of the surgery before scleral indentation were done according to the surgeon’s discretion.

**Fig. 1:**
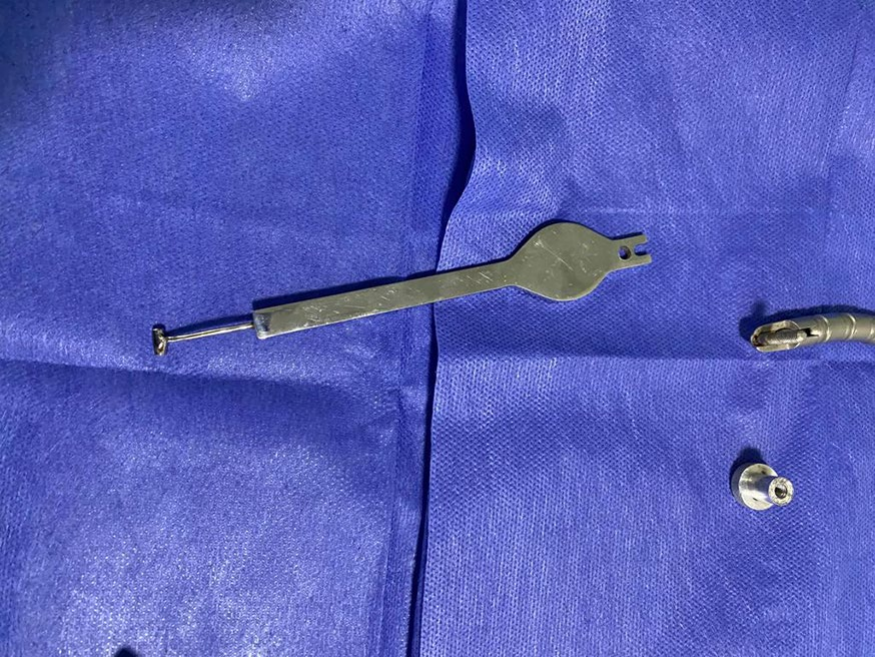
The tip of a thimble scleral indenter is welded to the support for the Leyla retractor system

**Fig. 2:**
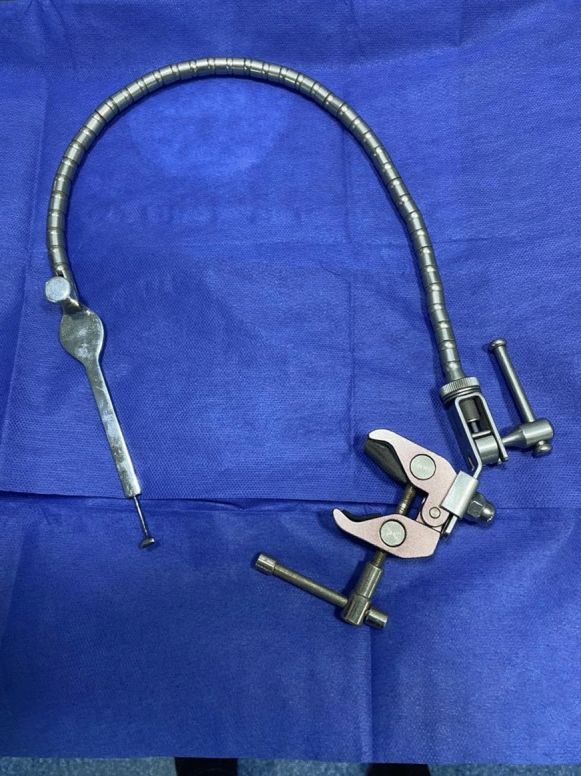
The Leyla retractor system with the attached scleral indenter in assembled

**Fig. 3:**
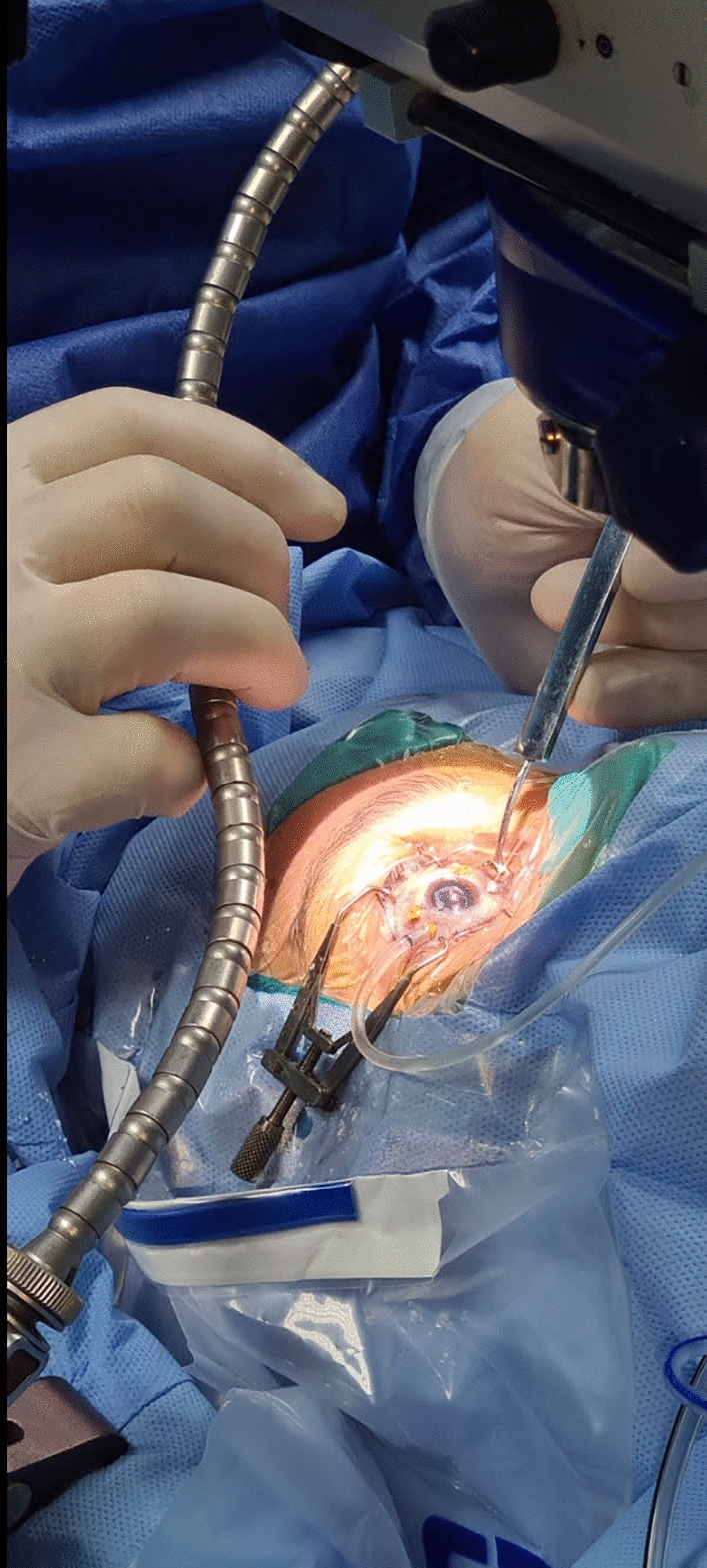
Intraoperative appearance of a case in the (Baha) indentation group showing the (Baha) technique. The surgeon is adjusting the position of the self-retained Leyla retractor system to achieve a satisfactory indentation

**Fig. 4:**
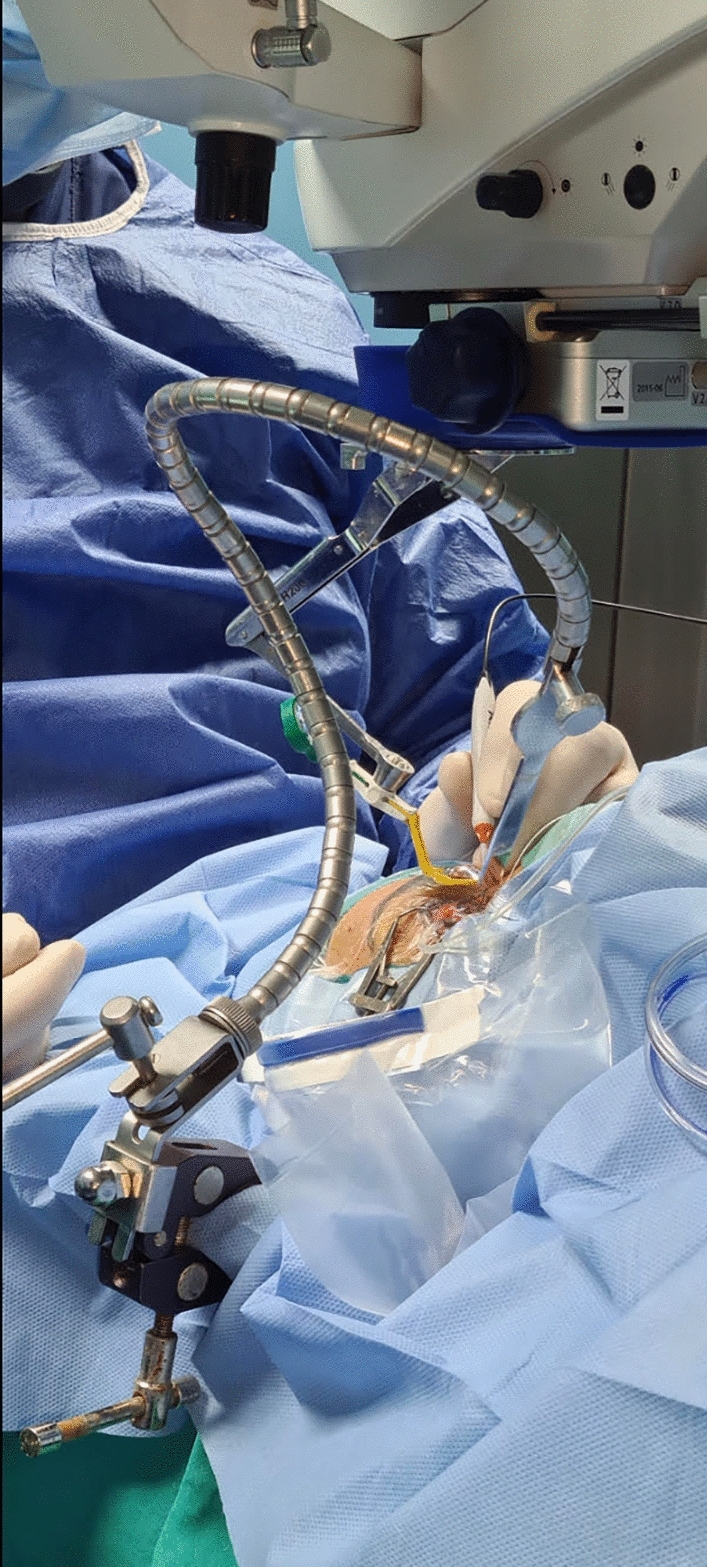
Intraoperative appearance of a case in (Baha) technique group using the self-retained Leyla retractor system while the Resight is in place

For the scleral indentation, cases in the first group had the (Baha) technique done. In which the tip of a thimble scleral indenter was welded to the support for the Leyla retractor system (B. Braun Medical Inc., Bethlehem, Pennsylvania, United States) (Figs. 1 and 2) was used for self-retained indentation. The fixation base of the retractor was fixed to the operating table over the sterile drape (Supplemental digital content 1). The operating surgeon insinuated the scleral indenter into the conjunctival fornix and adjusted the flexible arm to achieve a satisfactory scleral indentation while visualizing the fundus in the microscope and using a hand-held 23-gauge endo-illumination probe. The assistant then locked the wing screw to keep the scleral indenter in place. The surgeon used one hand to hold the endo-illumination probe and the second hand to hold the cutter or any other instrument (The surgical technique is shown in Figs. 3 and 4).

For cases in the second group, the surgeon performed bimanual surgery by holding a muscle hook in one hand to perform self-indentation and the cutter or any other instrument in the second hand.

For both groups, the shave mode was used during the removal of the peripheral vitreous while performing the scleral indentation. This mode uses a high cutting rate and lower vacuum level which allows safer removal of the peripheral vitreous in a controlled fashion while minimizing the risk of accidental cutting of the retina.

A video recording was done for all the cases. The videos were reviewed by another vitreoretinal consultant (I. A.) and analyzed to collect data about the (indentation duration) defined as the time in seconds between the first appearance of a hump due to scleral indentation in the recorded video until its final disappearance. In addition, the number of cases in the conventional indentation group in which readjustment of the direction of the endo-illumination to improve the illumination level and the number of adjustments done in every case, and the occurrence of any iatrogenic breaks or accidental touches of the crystalline lens in phakic eyes were recorded.

### Statistical analysis

The demographic, preoperative and intraoperative data of the two groups were compared using IBM SPSS software package version 20.0. (Armonk, NY: IBM Corp). The normality of the distribution of the variables was verified by the Kolmogorov-Smirnov test. Comparisons between groups were done for categorical variables using the Chi-square test (Fisher or Monte Carlo), and for normally distributed quantitative variables the student t-test was used. The significance of the obtained results was judged at the 5% level. The correlation between the vertical palpebral fissure height and the indentation duration will be calculated using the Pearson correlation coefficient (r).

## Results

The current study included 60 eyes of 60 patients with a mean age of 59.6 ± 9.8 years. Twenty-six (43.3%) of the included patients were females. Vitrectomy surgery was performed on the right side in 30 eyes (50%). Twenty-one of the included eyes were phakic and 21 were pseudophakic (including the cases with phacoemulsification done during the surgery) representing 65% and 35% of the included eyes, respectively. The mean vertical palpebral fissure height was 20.8 ± 9.5 mm. The patients were randomized on a 1:1 basis into two groups. The first group cases had the scleral indentation done during the pars plana vitrectomy surgery using the (Baha) indentation technique with the aid of the Leyla self-maintained retractor system. On the other hand, patients in the second group had conventional bimanual pars plana vitrectomies done with the use of a chandelier endo-illumination and self-indentation using a muscle hook. The features of the two study groups are summarized in Table 1.

**Table 1:** Features of the study groups

	(Baha) indentation	Conventional indentation	*P* value
Age (years)	56.7 ± 10.1	63.2 ± 9.5	0.91
Female no. (%)	12 (40%)	14 (46.7%)	0.87
Right eye no. (%)	16 (53.3%)	14 (46.7%)	0.85
Pseudophakic	11 (36.7%)	10 (33.3%)	0.94
Phakic	19 (63.3%)	20 (66.7%)	0.89
Phacoemulsification during the surgery	3 (10%)	2 (6.7%)	0.68
Palpebral fissure height (mm)	20.2 ± 11.3	21.4 ± 8.3	0.83

In the included eyes, multiple retinal breaks were found in 20 eyes (33.3%). And a giant retinal break was found in two eyes (6.6%). The primary retinal break was present in the superior quadrant in 30 eyes (50%), in the temporal quadrant in 13 eyes (21.7%), in the inferior quadrant in 8 eyes (16.7%), and in the nasal quadrant in 9 eyes (15%). The number of eyes with multiple breaks and the primary break locations of the two study groups are summarized in Table 2.

**Table 2:** The number of eyes with multiple breaks and the primary break locations no (%)

	(Baha) indentation			Conventional indentation			*P* value
	Phakic	Pseudophakic	Total (%)	Phakic	Pseudophakic	Total (%)	
Multiple breaks	7	5	12 (40)	4	4	8 (26.7)	0.27
Giant retinal break	1	0	1 (3.3)	1	0	1 (3.3)	1.0
Primary break location							
Superior	10	6	16 (53.3)	10	4	14 (46.7)	0.72
Temporal	4	2	6 (20)	4	3	7 (23.3)	0.63
Inferior	2	1	3 (10)	3	2	5 (16.7)	0.45
Nasal	3	2	5 (16.7)	3	1	4 (13.3)	0.5

The mean indentation time in the (Baha) technique cases was 618 ± 87 s. It was longer in the conventional indentation cases with a mean of 696 ± 72 s. However, the difference was not statistically significant (*p* = 38).

In cases of the conventional indentation group, readjustment of the chandelier endo-illumination was required in 19 cases (63.3%). The mean number of readjustments was 4.2 ± 3.6. Accidental touches of the crystalline lens and iatrogenic retinal breaks did not happen in any case in either group.

There was a positive correlation between the vertical palpebral fissure height and the indentation duration both for the (Baha) technique cases (*r* = 0.58) and the conventional indentation cases (*r* = 0.42).

Recurrent retinal detachment occurred in three cases in the (Baha) technique group (10%) and two cases in the conventional indentation group (6.7%). All the cases that had recurrent retinal detachment had reopened breaks after sulfur hexafluoride (SF6) gas tamponade was used in the initial surgery. All these cases had the retina successfully repaired after reoperation done within 2 weeks from the initial surgery with drainage of subretinal fluid, adding more laser shots around the retinal breaks and silicone oil injection. Scleral indentation during the second surgery was done using the same technique used in the first one.

## Discussion

Scleral indentation is a step of great importance during vitreoretinal surgeries. It can be done by self-indentation while using a chandelier endo-illumination. Alternatively, it can be done by the assistant while the surgeon is holding an endo-illumination light probe. However, this requires a skilled assistant and good coordination with the surgeon.

The use of an endo-illumination light probe has several advantages over chandelier endo-illumination. It allows the use of variable illumination techniques including direct illumination, specular illumination causing transparent surfaces to glow where light is shone at a critical angle, or retro-illumination [12].

In addition, the 23 gauge endo-illumination probe would give a brighter illumination compared to the 25-gauge endoilluminator due to better light transmission and larger surface area of the fiber optic [12].

Moreover, the chandelier endo-illumination gives a fixed more distant illumination which makes it more difficult to identify the dissection planes and to see transparent structures such as the vitreous or the epiretinal membranes (ERM), and may cause more glare after fluid-air exchange compared to focal illumination from light probes [12].

Finally, the heat buildup may occur in the steadily illuminated chandelier [13] and Shadows of the instruments crossing the path of the light may worsen the view.

Several types of chandelier illumination are available including Eckardt 25-gauge “twin light” chandelier illumination system, which provides homogenous lighting and fewer shadows than with single fibers [7], 25-gauge Tornambe Torpedo (Insight instruments, Stuart, FL), BrightStar (DORC, Zuidland, the Netherlands), Photon Light Source (Synergetics Inc., St Charles, MO) which uses a brighter xenon light source, or the light systems integrated into vitrectomy machines such as Constellation (Alcon, Fort Worth, Texas, USA), which is the one available in our hospital. A self-retaining 27-gauge chandelier endoilluminator was introduced in 2007 by Oshima [9], and 27-gauge twinlight chandelier illumination system was then introduced by Eckardt [8]. In addition, a 30-gauge dual fiber chandelier (Synergetics Inc.,St. Charles, MO) is available [14].

We observed that the (Baha) technique enabled a safe and effective scleral indentation. However, it was more difficult to perform the indentation in eyes with small palpebral fissures and in the nasal quadrant in eyes with deep sunken globes or high nasal bridges.

The Leyla retractor was previously used in several surgical techniques on other parts of the human body. It enables free movement of the scleral indenter to allow the surgeon to place it easily at the needed position. It can be fixed easily and quickly at the required position using a wing screw. This will allow a gentle, even scleral indentation while the surgeon is given the freedom to use both hands.

In addition, we observed that in the (Baha) technique it was possible to change the position of the indenter easily by unlocking the wing skew and changing the indenter position without removing the Resight objective lens.

We observed that insinuating the scleral indenter in the conjunctival fornix with the concavity of the indenter facing away from the globe, i.e., to use it as a rotator rather than an indenter of the globe provided better exposure of the peripheral retina.

We did not observe any major differences in the safety and efficacy of the indentation done in pseudophakic and phakic eyes using the (Baha) technique.

There are several disadvantages to the current work. The study included a relatively small number of cases. All the surgeries were performed by a single surgeon and in a single center. The technique was evaluated only for a single indication. And it was not evaluated in children.

## Supplementary Information

Below is the link to the electronic supplementary material.

Supplementary file1 (MP4 35,440 KB)

## Data Availability

The authors confirm they have full access to the data presented in the current manuscript and can provide them upon request with a reasonable cause.
